# DORIS: Personalized course recommendation system based on deep learning

**DOI:** 10.1371/journal.pone.0284687

**Published:** 2023-06-02

**Authors:** Yinping Ma, Rongbin Ouyang, Xinzheng Long, Zhitong Gao, Tianping Lai, Chun Fan

**Affiliations:** Computer Center, Peking University, Beijing, China; Beijing Normal University, CHINA

## Abstract

Course recommendation aims at finding proper and attractive courses from massive candidates for students based on their needs, and it plays a significant role in the curricula-variable system. However, nearly all students nowadays need help selecting appropriate courses from abundant ones. The emergence and application of personalized course recommendations can release students from that cognitive overload problem. However, it still needs to mature and improve its scalability, sparsity, and cold start problems resulting in poor quality recommendations. Therefore, this paper proposes a novel personalized course recommendation system based on deep factorization machine (DeepFM), namely **D**eep Pers**O**nalized cou**R**se Recommendat**I**on **S**ystem (**DORIS**), which selects the most appropriate courses for students according to their basic information, interests and the details of all courses. The experimental results illustrate that our proposed method outperforms other approaches.

## Introduction

With the wave of informatization, more and more colleges and universities have built their online learning platforms and shared offline courses here. Students can choose suitable courses from the platform to study conveniently. However, students must spend significant time selecting their preferred courses when faced with many courses. How to enable students to choose appropriate courses quickly has become a challenging issue for many colleges’ and universities’ online education platforms. In recent years, recommendation technology has achieved remarkable results in many fields, such as product recommendation in shopping malls, video recommendation in online playback platforms, etc. Therefore, how to use recommendation technology to assist students in choosing courses suitable for them has gradually become a popular field.

Recommendation technology has undergone many improvements over the past few decades. The traditional methods like content-based recommendation [1], collaborative filtering [2, 3] and mixed recommendation [4, 5] have been widely used in course recommendation, and deep learning techniques have also been applied to improve the course recommendation quality [6–10]. However, despite the unprecedented achievements in the course recommendation field, many very challenging problems could be solved.

For students, on the one hand, course recommendation suffers from a severe cold-start problem. Newly enrolled students only have basic information like department and major but need historical records on course selection. Therefore, it is difficult for classical methods such as collaborative filtering [11], content-based recommendation [12], and others to recommend courses accurately for the difficulty of modeling students’ interests [13]. On the other hand, students do not select courses based entirely on their interests, and they will find a balance between multi-objectives such as the course load, the difficulty of maintaining a high GPA, etc.

As for courses, they generally have enormous attributes, such as course introduction, prerequisite courses, credits, etc. Students can generally decide whether to take a course after fully understanding it. The various contents of the course are the only channel for students to understand the course. Therefore, fully modeling the course information is essential to the course recommendation. However, the text features of the course are more challenging to model than the attribute features, which is a very challenging problem in the course recommendation.

In this paper, we propose a **D**eep pers**O**nalized cou**R**se recommendat**I**on **S**ystem based on deep factorization machine (**DORIS**) that utilizes DeepFM to model the correlation between students and courses based on their features. In addition, we also explore the effectiveness of the course’s textual features by TextRank [14] transforming text features into a semantic representation which is easier for DeepFM to use.

The contributions of this paper can be summarized as follows:

- Improving the traditional methods to obtain students’ interests and their potential interests through deep learning networks.
- Proposing using TextRank and PCA to model the course’s textual feature.
- The AUC of our recommendation method is 0.969, much higher than the baselines.

The remainder of this paper is organized as follows. Section 2 provides an overview of the literature on the course recommendation system. Section 3 describes the recommendation methods. Section 4 discusses the experimental setup and results of the algorithm. Finally, section 5 concludes the research findings and discusses future work.

## Related work

Course recommendation is a hot research field and attracts many researchers’ interests. This section introduces the two mainstream methods for the course recommendation field: Traditional Course Recommendation and Deep Learning Based Course Recommendation.

### Traditional course recommendation

Course recommendation is one of the research hotspots in education. Researchers worldwide have put forward many methods in course recommendation [15]. There are three mainstreams of traditional personalized recommendation algorithms: content-based algorithm [1], collaborative filtering(CF) based algorithm [3, 16], and hybrid-based algorithm [2, 5, 17–21]. These algorithms have their characteristics, advantages, and suitable scenarios.

Content-based recommendation algorithm focuses on the feature description of users and items [22], and the recommendation results are well interpretable but are very similar to the items that users have displayed and explicit feedback, lacking diversity. The content-based recommendation was first used in an information retrieval system. As a result, many information retrieval and filtering methods can be used in content-based recommendation systems. The processing step of Content-based recommendation generally includes item representation, profile learning, and recommendation generation. For example, Morsomme et al. [1] proposed a Latent Dirichlet Allocation statistical model to fit a topic model; it can predict students’ academic interests and grades that the students will obtain in the course based on their transcript and recommend 20 courses that best match the student’s academic interest.

The recommendation algorithm based on collaborative filtering is the most widely used algorithm [23]. CF is an efficient information filtering technology in a personalized recommendation system. It can filter and analyze the collected information to analyze users’ interests and improve the quality of the information recommendation. This approach is based on the assumption that users have similar preferences if they have similar ratings for the same items [24]. Moreover, CF-based recommendation algorithms can be divided into memory-based and model-based CF recommendation algorithms. Memory-based CF algorithms can be further divided into user-based and item-based CF recommendation algorithms depending on the different objects [25]. Besides, Khorasani et al. [2] outline a Markov-based CF model by using the sequence of courses in each semester to recommend courses to students. In addition, Huang et al. [3] have put forward a cross-user-domain collaborative filtering algorithm to predict the top t optional courses with the highest predicted scores for one student by using the course score distribution of the most similar senior student.

However, both content-based and collaborative filtering-based algorithms face a cold start problem in the first stage of processing [26]. Therefore, a Hybrid-based recommendation algorithm was proposed to leverage this problem. Hybrid-based algorithms mix multiple technologies to compensate for each other’s shortcomings. The mixing method includes simple weighted fusion, switching, and mixing of recommendation results. Hybrid-based recommendation system attempts to use complementary advantages to create a system with higher overall performance and robustness [4]. For example, Nafea et al. [5] proposed a hybrid approach that combines collaborative filtering and item content filtering to achieve personal course recommendations. In recent years, the hybrid recommendation method using a knowledge graph to represent context information has attracted the attention of scholars. In the course recommendation, Xu et al. [27] fused with knowledge graph and collaborative filtering to increase the recommendation performance at the semantic level. Furthermore, they introduce a knowledge graph to establish the association between courses with which learners have interacted and courses with which they have not. In this way, they solved the cold start problem caused by data sparsity and missing.

### Deep learning based course recommendation

Deep learning is a machine learning algorithm that uses a multi-layer structure to learn and extract high-level features from raw data automatically. Hinton put forward the concept of deep learning in 2006 [28]. Then in the following ten years, many theories and deep learning methods gradually developed and broke out [29–36].

Due to the excellent performance of deep learning in the fields of natural language processing [37, 38], computer vision [39, 40] and speech recognition [41], there have been studies on the use of deep learning technology to enhance course recommendation results. In addition, researchers found that deep learning methods can overcome the shortcomings of traditional approaches, such as accuracy, sparsity, and scalability. Dien et al. [8] proposed to use multi-layer perceptron to build a student’s performance prediction model with entrance English testing grades, activity incentive grades, etc. However, this method does not consider the high-level user and course features. Li et al. [10] proposed a DECOR module based on deep learning, which consisted of two parallel sub-modules, both are feed-forward neural networks (FFNN). One is to capture high-level user behavior features, and the other is to capture high-level course attribute features, then the module outputs the predicted probability that the user will choose the course. Nevertheless, DECOR can not deal with sequence, concurrency, constraints, and concept drift problems. Wong [6] proposed Long Short-Term Memory (LSTM) Recurrent Neural Networks to overcome the difficulty of problems as mentioned above. However, none of the methods mentioned above deal with the course introduction to improve the course portrait or integrate students’ course selection information into the network to improve the neural network’s performance. Therefore, this paper applies deep learning to the course recommendation system, constructs the student portrait by combining the data of students’ history courses, grades, and majors, uses the course department, average score, and the introduction of courses to construct the course portrait, and finally use the deep neural network to analyze the students’ interests for the recommendation. Our proposed method is based on enrollment data and no prior syllabus knowledge.

## Methods

**Fig 1:**
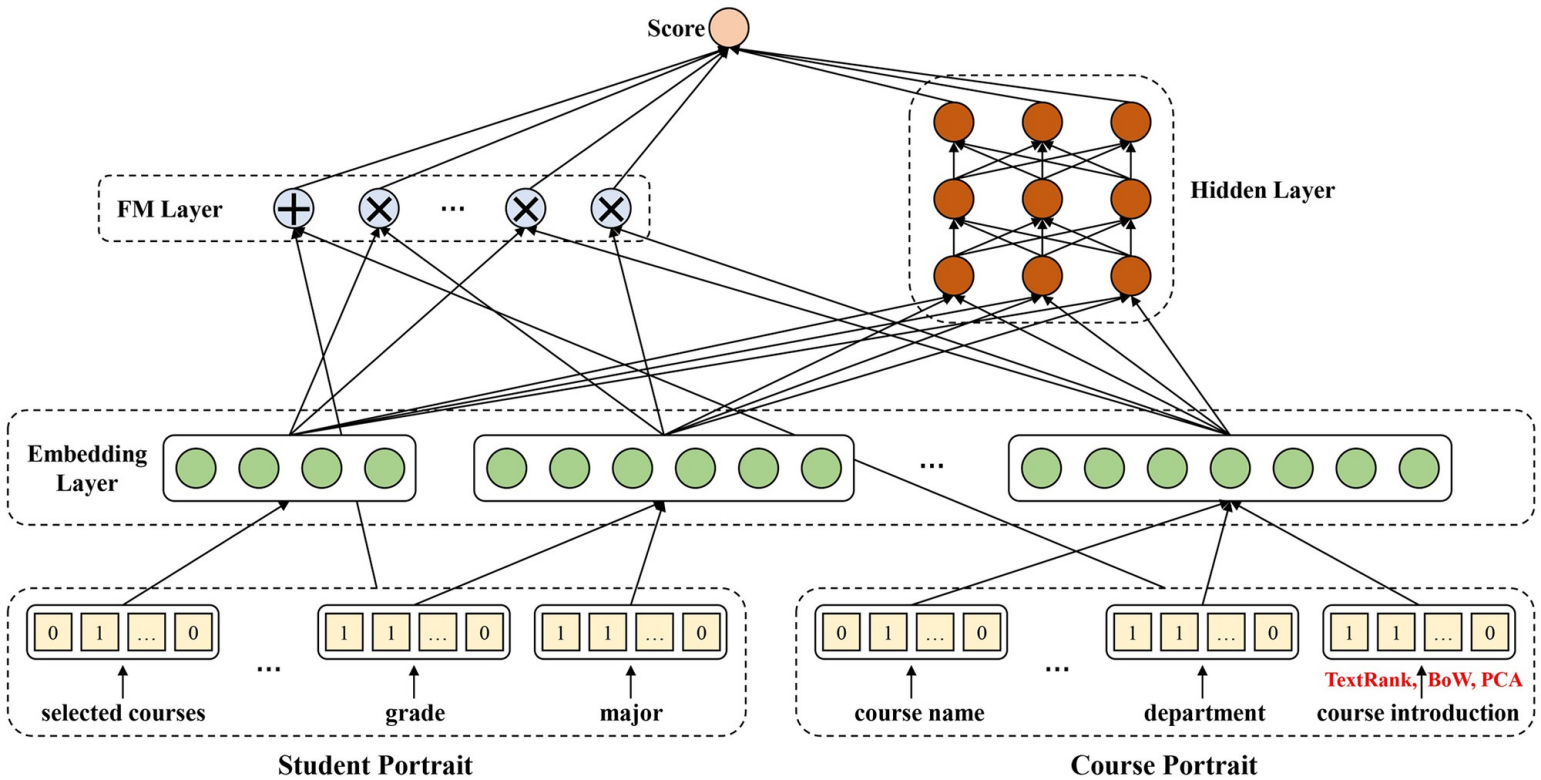
The overall architecture of DORIS has three main layers. The bottom layer is the input containing the student and course portrait. The middle layer transforms the original input into dense representations. The upper layer includes FM (left) and DNN (right). Finally, the scores from FM and DNN are integrated into one score.

Our proposed method is based on Deep Factorization Machine (DeepFM), which can extract low-order and high-order features simultaneously. In this section, we first introduce the architecture of DORIS. Then, we will display the details of constructing a student portrait, including basic information and historical course records. Finally, we will show the course portraits and how to process the textual features by TextRank and PCA. The overall architecture of DORIS is shown in Fig 1.

### Problem formulation

The course recommendation task is to measure the probability of click among a set of candidates $C={\left\{c_{i}\right\}}_{i=1}^{N}$ for a user *u* ∈ *U*, where *C* is all courses in the platform, and *u* is a student who wants to find proper courses for learning. we are required to find a function f(.,.):U×C→R, which can be formulated as:

$$\begin{array}{c} f^{⋆}=\underset{f}{\text{max}\;}E\phi \left(Y,F\left(u,C\right)\right) \end{array}$$ (1)

where *ϕ* is a evaluate metric such as AUC, LogLoss, etc. $F\left(u,C\right)={\left\{f\left(u,c_{i}\right)\right\}}_{i=1}^{N}$ is the set of probabilities for all courses, and *f*(*u*, *c*_*i*_) is a model that we need to optimize, and its output denotes the score of the course *i* for user *u*. Generally, the higher *f*(*u*, *c*_*i*_), the more likely *c*_*i*_ is to be selected by *u*. $Y={\left\{y_{i}\right\}}_{i=1}^{n}$ is a set of scale with *y*_*i*_ representing *u* selecting *c*_*i*_ or not.

The ultimate goal is to train a recommendation model with a set of labeled <user, course> pairs *Ω* = *ω*_*u*_ where *ω*_*u*_ = {*u*, *C* = {*c*_*i*_}, *Y* = {*y*_*i*_}|0 < *i* ≤ *N*_*u*_, *y*_*i*_ ∈ {0, 1}}. Under the formulation, the recommendation model is optimized by minimizing the empirical loss over the training data as:

$$\begin{array}{c} L\left(f\right)=\frac{1}{\left|Z\right|}\sum_{u,C,Y}\ell \left(Y,F\left(u,C\right)\right). \end{array}$$ (2)

where *ℓ* is a loss function such as cross-entropy, which is an intermediate proxy for optimizing the none-differential metric *ϕ*, and Z is a normalizing factor.

### Deep personalized course recommendation system

Let *X* = [*u*, *c*] denote the input features of DORIS where *u* = [*u*_1_, *u*_2_, …, *u*_*n*_], *c* = [*c*_1_, *c*_2_, …, *c*_*m*_] and *n*, *m* stands for the feature size of the user and course respectively. It is worth noting that every element in *u* and *c* can be continuous and categorical optionally. The details of constructing the user and course features will be introduced in the next section. The DORIS is based on DeepFM, composed of two parts: the DNN component and the FM component. The final prediction DORIS is based on the output of both components:

$$\begin{array}{c} y=\sigma (y_{dnn}+y_{fm}) \end{array}$$ (3)

where *σ* is an activation function which is defined as $\left(x\right)=\frac{1}{1+\text{exp}\left(-x\right)}$. *y*_*dnn*_ and *y*_*fm*_ are the output of DNN component and FM component. *y* ∈ (0, 1) is the predicted CTR. The higher *y*, the greater the possibility of the course *c* being selected by user *u*.

#### DNN component

The DNN component is a deep neural network that aims at learning the high-order interactions between features. Generally, *X* consists of continuous and sparse values, and the input size of *X* can be enormous. Therefore, an embedding layer is introduced to compress the input *X* into a low-dimensional dense vector, and the output of the embedding layer can be denoted as:

$$\begin{array}{c} E=[e_{1},e_{2},\ldots ,e_{N}] \end{array}$$ (4)

where *e*_*i*_ denotes the embedding of *i*-th field and *N* is the number of field. Then, *E* is fed to the deep neural networks and can be viewed as the 0-th output of DNN, and the process of DNN can be denoted as:

$$\begin{array}{c} o_{l}=\mu \left(W_{l}o_{l-1}+b_{l}\right) \end{array}$$ (5)

where *μ* means activation function, such as *tanh*, *relu* and etc. *o*_*l*_, *W*_*l*_, *b*_*l*_ are the output, model weight and bias of *l*-th DNN layer. Finally, we get the high-order interaction representation *o*_*L*_ where *L* is the number of a hidden layer of DNN, and the prediction of DNN is:

$$\begin{array}{c} y_{dnn}=\sigma \left(W_{dnn}o_{L}+b_{dnn}\right) \end{array}$$ (6)

where *μ* is sigmoid function, and *W*_*dnn*_, *b*_*dnn*_ are the learnable parameters of DNN’s prediction layer.

#### FM component

Rendle et al. [42] first proposed the factorization machine method for the recommendation field. The FM method can effectively learn the first-order feature interaction and the second-order feature interaction. Specifically, the parameter of interaction feature *i* and *j* is the inner product of their corresponding latent vector *V*_*i*_ and *V*_*j*_. The output of the FM component is defined as:

$$\begin{array}{c} y_{fm}=W_{fm}^{0}+\sum_{i=1}^{N}W_{fm}^{i}X_{i}+\sum_{i=1}^{N}\sum_{j=i+1}^{N}\left\langle V_{i},V_{j}\right\rangle \left\langle X_{i},X_{j}\right\rangle \end{array}$$ (7)

where $\langle V_{i},V_{j}\rangle =\sum_{f=1}^{k}V_{i,f}V_{j,f}$ is the inner product of *V*_*i*_ and *V*_*j*_, and *k* is the dimension of latent vector. $W_{fm}^{*}$ denotes the learnable parameters of the FM component.

In this way, the second-order interaction parameters can be learned without the constraint of the co-occurrence of both features. Therefore, the FM method can thoroughly learn the interaction between features.

### Student and course portrait

One of the critical factors in a recommendation system is to mine a variety of compelling features for the user and items. The student portrait refers to mining and extracting students’ labels on different attributes from various data generated by students, such as their grades, department, major, selected courses, etc. The course portrait refers to labels with different attribute characteristics of courses, such as course number, introduction, and prerequisites. The accurate student portraits and course portraits directly affect the accuracy of the personalized course recommendation system, thus affecting the user experience of students.

#### Student portrait

According to the means of obtaining student portrait, the construction methods of student portrait can be divided into two categories which can be summarized as below:

- **Basic student features** can be directly fetched from their registered information, including major, grade, semester, and other information. This kind of information is critical for course recommendation. For example, there is no doubt that students will select the required course of the corresponding major.
- **High-level student features** can be induced from students’ historically selected course records. For example, many advanced courses require prerequisites that the historically selected courses can explicitly indicate. In addition, the average score of all taken courses stands for a student’s learning ability, and students should be recommended courses matching their capacities.

#### Course portrait

The course portrait is mainly composed of basic information (e.g., course name, id, college, type, grade, prerequisite, and introduction) and high-level features like the average score of all students.

Course introduction is a brief statement that introduces the course content and teaching plan. In addition, it contains the characteristic information of the course, which can extract the course label information to enrich and enhance the course portrait to make the course recommendation network recommend courses to students more accurately.

The course introduction consists of several natural language sentences, and the course name can be regarded as the shortest course introduction. In this topic, we splice the course name and the course introduction together as the course introduction.

From a general perspective, the course introduction can not be used directly in DORIS, and it should be transformed into a real-value feature for better understanding by DORIS. This paper uses a bag-of-word to change the course introduction into a one-hot vector that DORIS can understand.

As is known to all, the recommendation system should return the result as soon as possible for a good user experience. However, the dimension of course introduction is tremendous, leading to unaffordable and time-consuming DORIS. Therefore, compressing the course introduction feature into an acceptable size is very important. To overcome these difficulties, we first take the TextRank [14] approach to select important words representing the course introduction’s semantics.

The basic idea of the TextRank algorithm originates from the PageRank algorithm: dividing the text into a sequence of words that are not stop-words, establishing a graph model, and using the voting mechanism to sort the crucial components in the text. After that, the keywords and abstracts in the text are extracted.

The first step is to construct a graph *G* = (*V*, *E*), where *V* and *E* are the node set and edge set for graph *G*. In TextRank, *V* is composed of the word sequence of all course introductions with *n* words: [*v*_1_, *v*_2_, …, *v*_*i*_, …, *v*_*n*_], and the edge relationship is the co-occurrence of words in a limited context window. Finally, the weight of each word *v*_*i*_ at iteration *k* can be defined as:

$$\begin{array}{c} S{\left(v_{i}\right)}^{k}=1-d+d\times \sum_{j\in In(v_{i})}\frac{1}{\left|Out(v_{j})\right|}S{\left(v_{j}\right)}^{k-1} \end{array}$$ (8)

where *d* is a damping factor in avoiding dead ends. *In*(*v*_*i*_) stands for all nodes pointing to node *i*, and *Out*(*v*_*j*_) means the number of nodes that node *j* point to. After *K* steps of iteration, we can obtain the top-N keywords (TopN-Word set). The words in the TopN-Word set will be kept, and the left will be abandoned.

The TextRank method can reduce the dimension of the course introduction feature to some degree, but the size is still enormous for DORIS. Therefore, we further adopt the principle component analysis (PCA) [43] to reduce the dimension of the course introduction feature.

## Experiments

In this section, we first introduce the details of dataset construction. Then, we show the evaluation metric used in our paper to measure the performance of different methods. After that, we depict the hyper-parameters setting in our experiment and display the baselines that DORIS compares with. Finally, we show the results of all methods and analyze their performance.

### Dataset

This paper collected an anonymized dataset from Peking University between 2014 and 2021 to analyze students’ behavior. There are 4568 students, 5591 courses, and 208949 actual course enrollments. A course enrollment means that the student was enrolled up to the end of the semester. The course data consists of 53 departments of Peking University, such as Archaeology and Museology, Electronics Engineering and Computer Science, College of Engineering, and Guanghua School of Management. Each course has a brief introduction, and the course name can optionally be regarded as an introduction if the course introduction is missing. There are 2107 courses with prerequisites in our dataset; they are written in natural language texts by the teacher of the course. In the actual course selection, the course selection is not limited according to these prerequisites; they are just suggestions for students’ course selection. Table 1(a) presents some examples of student data. Each student has the characteristics of student number, year of enrollment, education background, and major. Table 1(b) presents some examples of course data. Each course has the characteristics of course number, course name, college, course type, grade, prerequisite, and introduction. Table 2 present some example of course selection data.

**Table 1:** Examples of student and course data.

(a) Example of student data.			
**StudentId**	**EnrollmentYear**	**Education**	**Major**
1	2013	本科 Undergraduate	历史学 History
2	2013	本科 Undergraduate	市场营销 Marketing
3	2014	本科 Undergraduate	物理学 Physics
4	2014	本科 Undergraduate	地理信息科学 Geographic Information Science
13	2014	本科 Undergraduate	金融学 Finance
14	2014	本科 Undergraduate	-
3923	2019	研究生 Postgraduate	-

(b) Example of course data						
**CourseID**	**Name**	**College**	**Type**	**Grade**	**Prerequisite**	**Introduction**
1	大数据分析中的算法 Algorithms for Big Data Analysis	数学科学学院 School of Mathematical Sciences	必修 Compulsory	3	-	大数据给数据分析和处理带来了… Big data brings…
2	黎曼几何中的比较定理 Comparison Theorems in Riemannian Geometry	数学科学学院 School of Mathematical Sciences	选修 Elective	3	代数拓扑初步 Preliminary Algebraic Topology	本课程中我们将学习完备的黎曼… In this course, we will learn the connection between…
7	贝叶斯理论与算法 Bayesian Theory and Computation	数学科学学院 School of Mathematical Sciences	选修 Elective	3	概率论,多元分析 Probability Theory, Multivariate Analysis	贝叶斯分析是统计学的一个重要分支… Bayesian analysis is an important branch of statistics…
353	优化和学习的模拟方法 Simulation Methods for Optimization and Learning	工学院 College of Engineering	-	-	-	本课程讲解了使用计算机模拟进行… This course explains the essentials of dynamic stochastic…
3981	加拿大法语文学 French Literature of Canada	外国语学院 College of Foreign Languages	选修 Elective	2	-	-
4193	高级体育训练(四) High-Level Sports Training (4)	-	全校必修 Compulsory	1	-	-

**Table 2:** Example of course selection data.

StudedntId	AcademicYear	Semester	CourseId	CourseName	CourseCollege	Score
1	13–14	1	1876	世界史通论 A General Survey of World History	历史学系 Department of History	59
1	13–14	1	4658	文科计算机基础（上） Fundamentals of Computer for Arts (1)	信息科学技术学院 School of Electronics Engineering and Computer Science	60
1	13–14	1	4360	美术概论 Introduction to Fine Arts	艺术学院 School of Arts	78
2	13–14	1	2632	经济学 Economics	光华管理学院 Guanghua School of Management	36
2	13–14	1	4158	足球 Football	体育教研部 Section of Physical Culture	93
1017	20–21	2	167	线性代数 (B) Linear Algebra (B)	国家发展研究院 National School of Development	-
1017	20–21	2	168	线性代数 (B)习题 Problem-Solving on Linear Algebra (B)	信息科学技术学院 School of Electronics Engineering and Computer Science	-
1652	18–19	1	4125	形势与政策 Events and Policies	马克思主义学院 School of Marxism	60
1652	18–19	1	4152	健美操 Aerobics	体育教研部 Section of Physical Culture	-
2694	19–20	1	196	高等代数（I）习题 Problem-Solving on Advanced Algebra (I)	数学科学学院 School of Mathematical Sciences	-
2694	19–20	1	260	音乐与数学 Music and Mathematics	数学科学学院 School of Mathematical Sciences	93
3700	20–21	1	1938	古典学导论 Introduction to Classical Studies	历史学系 Department of History	47
3700	20–21	1	2145	西方哲学（下） Western Philosophy, PartⅡ	哲学系 Department of Philosophy	40

As shown in the table above, students and courses have lots of missing information. For example, there are 149223 course-score records out of 208949 course-selection records. Statistics of missing data in all datasets are shown in Table 3.

**Table 3:** Statistics of missing data in the dataset.

Features	Student		Course			
	Education	Major	Type	Grade	Prerequisite	Introduction
Total	4568	4568	5591	5591	5591	5591
Missing	6	744	367	365	3484	348

The user and course portrait are combined as training items in training data construction. For example, a training or prediction item contains the following features:

(StudentID, EnrollmentYear, Education, Major, AverageScore) + (CourseID, CourseName, CourseCollege, AcademicYear, Type, Grade, Semester, Department, Score) + (Dimensionality Reduction of Processed Course Introduction, Processed Course Prerequisites).

There are 208949 items of entire course selection when making the training set, and then counterexamples are made according to the ratio of entire course selection data to false course selection data of 1:1, 1:20, and 1:40. The so-called counterexamples are the combination of students and courses that have not been selected. The proportion of positive and negative examples in the verification set is consistent with that in the training set.

In testing data construction, all students in the test set did not appear in the training set. Therefore, for each student in the test set, combine all 5591 courses with the student to form 5591 items, including positives and negatives. For inference, we first predict the score of 5591 items and rank all items based on the predicted scores in descending order. The top-N courses will be regarded as the proper courses for students.

### Evaluation metric

In this paper, **AUC** and **LogLoss** are adopted to measure the performance of baselines and DORIS. The details of two evaluation metrics are shown below:

- **AUC** is the abbreviation of **Area Under the Curve** and is a performance measurement for classification problems at various threshold settings. The higher the AUC, the better the model predicts the 0-class as 0 and the 1-class as 1.
- **LogLoss** indicates how close the prediction probability is to the corresponding actual/true value (0 or 1 in case of binary classification). The more the predicted probability diverges from the actual value, the higher the log-loss value.

### Experimental setting

In the DORIS, the latent dimension of FM is 8, and the DNN has MLP with three layers with a hidden size is 128. The dimension of the course introduction is set to 394. The training and evaluating batch size are set to 2048. The activation function is relu, and the dropout rate is set to 0.2. We use the Adam [44] to optimize our model with the learning rate of 0.0005, and two momentum coefficients are set to 0.9 and 0.999, respectively. All the models are trained on the distributed platform with Linux system armed with AMD EPYC 7302 16-Core Processor, 503G Memory, 8 NVIDIA A100 GPU, and 12T Disk.

### Baselines

To comprehensively evaluate the performance of DORIS, we list some baseline approaches for comparison. The baselines are introduced as follows.

- **BPR** [45] is a generic optimization criterion BPR-Opt for personalized ranking that is the maximum posterior estimator derived from a Bayesian analysis of the problem.
- **LR** [46] uses features of ads, terms, and advertisers to learn a model that accurately predicts the click-through rate for new ads.
- **FM** [42] combines the advantages of Support Vector Machines (SVM) with factorization models, and it models all interactions between variables using factorized parameters.
- **DSSM** [47] is a new latent semantic model with a deep structure that projects queries and documents into a common low-dimensional space where the relevance of a document given a query is readily computed as the distance between them.
- **DECOR** [10] is a novel deep learning-based course recommender system that elaborately captures high-level user behaviors and course attribute features.

### Result

Table 4 reports the results of our models in comparison to the other reference methods. Again, it can be seen that our DORIS can achieve state-of-the-art results compared to other baselines.

**Table 4:** The results of proposed method and baselines.

Model	BPR	DSSM	LR	FM	DECOR	DORIS
AUC	0.1706	0.8717	0.8966	0.9106	0.9317	0.9693
LogLoss	34.1746	0.3917	0.3003	0.2250	0.1174	0.0349

From Table 4, it can be seen that the BPR model has the worst performance among all approaches. The main reason is that it does not use any valuable features besides student and course id. However, this also implies that abundant features play significant roles in the course recommendation system.

The DSSM significantly improves performance over BPR, but its performance is worse than that of LR and FM models. Since it is known that DSSM learns the representations of students and courses without any interaction between them, this leads to worse performance.

As we all know, the LR model can not learn the high-order feature interaction, whereas the FM model can address the problem of LR. As a result, FM performs better than LR. DECOR is a well-designed course recommendation system, and we can see that its performance is better than general recommendation methods such as BPR, LR, and FM.

In conclusion, DORIS can achieve the best results because it combines the benefits of deep neural networks and FM models. What is more, it makes full use of the course’s introduction and prerequisites. However, in the future, it is necessary to mine more useful features to further improve the performance of course recommendations.

## Conclusion

In this paper, we present DORIS, a DeepFM-based course recommendation system, which can not only make full use of basic information about students and courses but also model the historical course selection records of students and the introduction and prerequisite of course. Our proposed DORIS can achieve extraordinary results in the actual course recommendation scenario. However, DORIS also faces some challenging difficulties. First, the proposed methods can not solve the cold start problem, and we can address this problem by (1) requiring a user to provide more information; (2) leveraging transfer learning methods. Second, the text is encoded by PCA and TextRank, which do not have strong fitting abilities; we can make full use of capable encoders such as CNN [48], RNN [49] and Bert [50].

## Supporting information

S1 Dataset (ZIP)

S2 Dataset (ZIP)
